# Is Technology Present in Frailty? Technology a Back-up Tool for Dealing with Frailty in the Elderly: A Systematic Review

**DOI:** 10.14336/AD.2016.0901

**Published:** 2017-04-01

**Authors:** Iranzu Mugueta-Aguinaga, Begonya Garcia-Zapirain

**Affiliations:** ^1^Rehabilitation Service, Cruces Universitary Hospital, Plaza Cruces s/n, 48903, Barakaldo, Spain.; ^2^DeustoTech - Deusto Foundation, Avda Universidades, 24, 48007, Bilbao, Spain; ^3^Engineering Faculty, University of Deusto, Avda. Universidades, 24, 48007, Bilbao, Spain

**Keywords:** Frailty, kinect, exergaming, serious games, robots, virtual reality

## Abstract

This study analyzes the technologies used in dealing with frailty within the following areas: prevention, care, diagnosis and treatment. The aim of this paper is, on the one hand, to analyze the extent to which technology is present in terms of its relationship with frailty and what technological resources are used to treat it. Its other purpose is to define new challenges and contributions made by physiotherapy using technology. Eighty documents related to research, validation and/or the ascertaining of different types of hardware, software or both were reviewed in prominent areas. The authors used the following scales: in the area of diagnosis, Fried’s phenotype model of frailty and a model based on trials for the design of devices. The technologies developed that are based on these models accounted for 55% and 45% of cases respectively. In the area of prevention, the results proved similar regarding the use of wireless sensors with cameras (35.71%), and Kinect™ sensors (28.57%) to analyze movements and postures that indicate a risk of falling. In the area of care, results were found referring to the use of different motion, physiological and environmental wireless sensors (46,15%), i.e. so-called smart homes. In the area of treatment, the results show with a percentage of 37.5% that the Nintendo^®^ Wii™ console is the most used tool for treating frailty in elderly persons. Further work needs to be carried out to reduce the gap existing between technology, frail elderly persons, healthcare professionals and carers to bring together the different views about technology. This need raises the challenge of developing and implementing technology in physiotherapy via serious games that may via play and connectivity help to improve the functional capacity, general health and quality of life of frail individuals.

According to the World Health Organization (WHO), it is estimated that there are more than 605 million people over 60 years of age in the world. The proportion of elderly persons will go on increasing over the coming decades - by the year 2025 it is estimated that there will be 1,200 million elderly persons throughout the world and two out of every three will be living in developing counties [1].

Spain is currently one of the countries in the world with the highest life expectancy after Japan, but when one speaks of life expectancy in terms of good health, the situation worsens in relation to other countries such as France, Sweden, Australia and Japan. For this reason, it is important to highlight the fact that living longer is not always a synonym for good quality of life and health [2].

Human ageing is a process that is characterized by the gradual loss of physical and cognoscitive capacities, and maintaining functional independence until the end of one’s life has been and remains the most ambitious goal pursued by geriatrics [3].

Despite accounting for a large sector of the population, there are gaps in our knowledge with regard to elderly persons. Specifically, there is a group of elderly persons who are just on the limit, on the edge of decline - what is referred to after 70 years of age as a frail elderly person [4].

The frailty in elderly persons has witnessed exponential growth in research and clinical practice in recent years [5], with there being consensus about the definition of frailty as a deregulated (potentially correctable or improvable) situation in many biological systems, accumulation of deficits, a reduction in physiological reserve and proneness to a range of adverse events [6,7].

Numerous studies have suggested that frailty is a detector of functional decline and mortality [8-10], and there are those that also show that the prevalence of frailty increases significantly as age increases, from 3.2% at 65 to 16.3% at 80 and 23.1% at 91 years of age [11].

In the case of Spain, there are six longitudinal cohorts that pinpoint the prevalence of frailty in different proportions according to age groups and the criteria used to measure it (Fig. 1).

**Figure 1.**
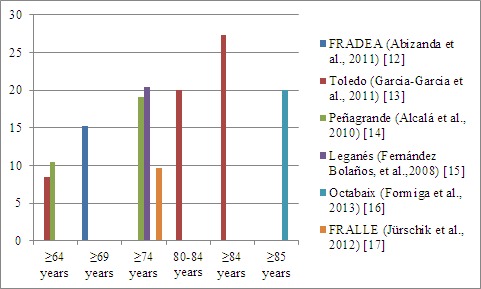
Prevalence of frailty in Spain. Data from cohorts of longitudinal aging studies in Spain.

According to the review by Ng et al., 2013 [18], the frailty requires a new approach based on the following reasons. Firstly, frailty is interconnected but may occur irrespective of any comorbidity or inability [19, 20]. Secondly, frailty is associated with a greater risk of hospital readmission, admission to a care home, a worse outcome following surgery, post-operative complications and a greater risk of falling, dementia, general morbidity and mortality. Thirdly, it is potentially reversible as a result of specific intervention such as rehabilitation and exercise [18].

Frailty occupies a prominent place worldwide, its being necessary to invest in improving quality of life and health in order to provide individuals with a longer, healthier life and thus be able to maintain an independent life for as long as possible - and therefore avoid frailty or at least reduce it.

Numerous clinical studies have advocated muscular strengthening and exercise as being beneficial even in frail elderly persons [21-24], both with a view to reducing the risk of falling and reducing general decline, while improving mobility and functional capacity [25].

Technology-based innovation will prove to be the driving force capable of turning the current situation around by offering new opportunities [26].

In an ageing society it is necessary to establish new alternatives that may somehow try to meet the needs of elderly persons while increasing their perceived quality of life. In this respect, new technologies have become a basic tool in our society.

The costs of an ageing society in the future will be unsustainable in terms of health care and social services, unless they can be organized via an approach that focuses on prevention and promotion of health within an integrated system. Technology-based innovation will prove to be the driving force capable of turning the current situation around by offering new opportunities [26].

Specialist literature contains many reviews of studies that include technology as support and tools that can be used to provide benefits to medical practice, thus taking into consideration new future challenges [18, 27].

## MATERIALS AND METHODS

By observing the major role played by technology in our modern-day society, we may ask the following question: to what extent is technology present in its relationship with frailty or, put another way, what technological resources are used to deal with frailty? We are interested in establishing which devices and programs have been developed thus far to help deal with the issue of frailty that are related to prevention, diagnosis, care and treatment, among other areas.

To respond to this question, we decided to conduct a search by way of a review from January 2005 to December 2015, in the course of which four databases were consulted:

- OVID Medline <1946 to December week 4 2015>
- EMBASE via OVID < 1974 to 2015 week 53>
- Web of Science (2005-2015): gathering together all the databases included: main Web of Science™ collection, Current Contents Connect^®^, Derwent Innovation Index^SM^, Inspec^®^, KCI-Korean Journal Database, MEDLINE^®^ and SciELO Citation Index
- IEEE Xplore (2005-2015)
- Filters: date (2005-2015), language (English).

The search was undertaken in accordance with the following two strategies, owing to the features of the databases selected:

1. Medline and EMBASE: these databases permit the use of descriptors and even expand on them, combining them always with natural language and using the following keywords:
  - As expanded descriptors: frail elderly, software, computers (hardware term was included), telecommunications, videogames.
  - As natural language: frailty, software, telecommunications, kinect, videogames, wii, exergame, exergaming, sensors, serious games, robots, virtual reality.
2. Web of Science and IEEE Xplore: conversely, these databases do not permit the use of descriptors, whereby exclusively natural language must be used using the following keywords:
  - As natural language: frailty, software, hardware, telecommunications, kinect, videogames, wii, exergame, exergaming, sensors, serious games, robots, virtual reality.

The following results were obtained from the review:

- OVID Medline <1946 to December week 4 2015>: n=130
- EMBASE via OVID < 1974 to 2015 week 53>: n=304
- Web of Science (2005-2015): n=271
- IEEE Xplore (2005-2015): n=136

**Figure 2.**
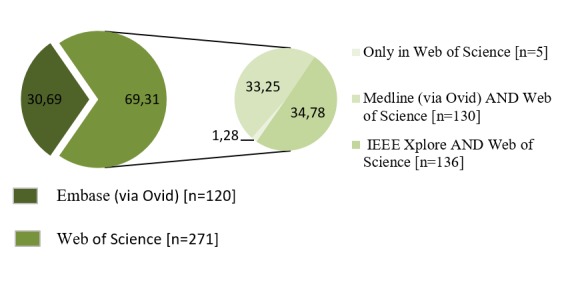
Percentage of results according to the data base that was reviewed.

**Figure 3.**
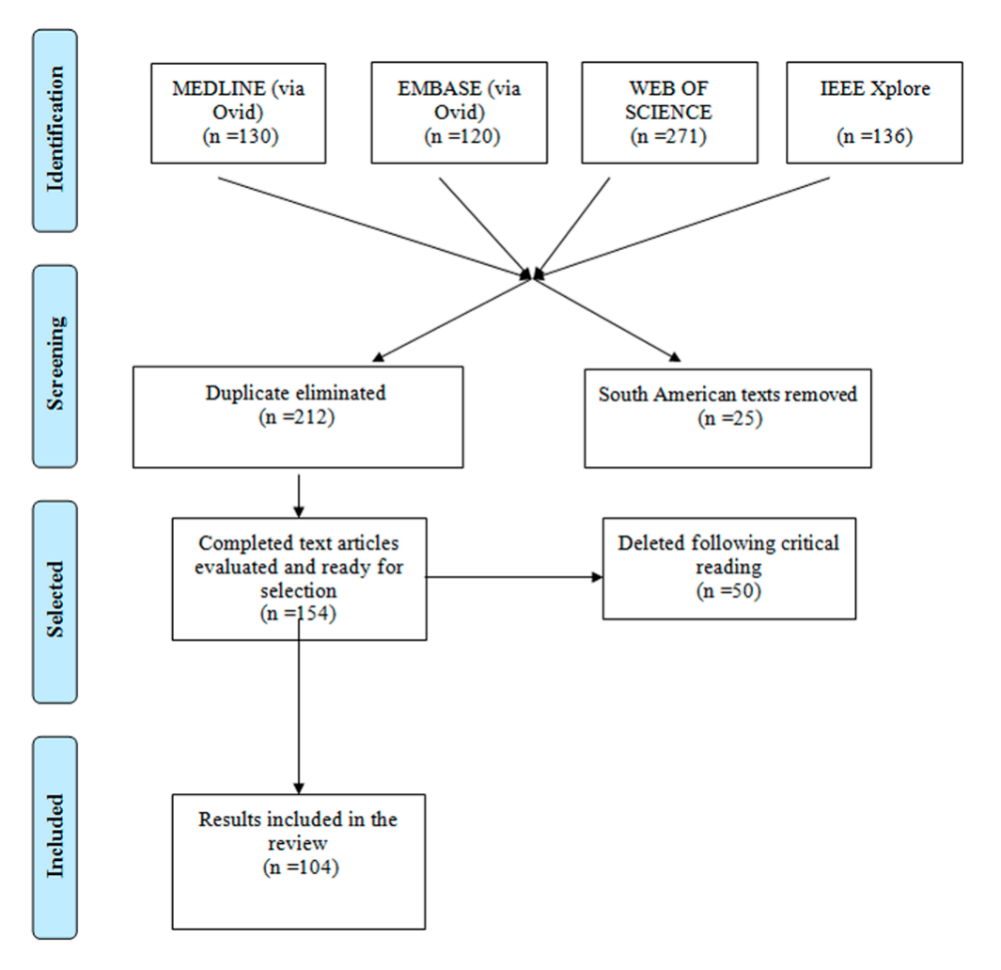
Flow Diagram. Strategy carried out in this review

Inclusion criteria: Articles that deal with programs and devices developed in relationship with frailty.

Exclusion criteria: Articles that do not deal with programs and devices developed in relationship with frailty.

The results were reviewed to control duplicates by noting that most papers in these databases could also be found in the Web of Science (Fig. 2).

For this reason, the decision was made to work with the results obtained from the Web of Science alongside those from EMBASE, as in this last-mentioned database can be found some European publications not found in the Web of Science. Following critical reading of the documents, the total number included in the review amounted to 104 documents (Fig. 3).

Two reviewers have inspected independently all titles and abstracts of all references obtained from the searching strategy. Abstracts of the articles selected have been evaluated to determine if they were in accordance to the inclusion criteria. The disagreements were solved by agreement and/or coordinated review. The final list of the selected studies has been revised separately by both authors.

The quality grades and scientific evidence level have been classified according to the criteria proposed by The Swedish Council on Technology Assessment in Health Care (SBU) [28].

**Figure 4.**
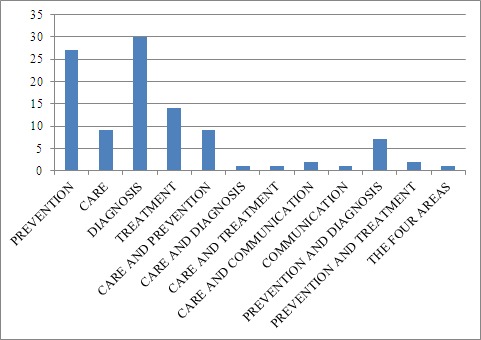
Results found between 2005-2015 for specific areas

The 104 documents were analyzed meticulously so as to classify their content. Thus, the following areas of work related to frailty were the ones mainly observed: prevention, diagnosis, care and treatment. These in turn require some type of hardware or software and in fact both in most cases. Studies exist in which the research covers one or more areas of care (Fig. 4), although this review has tended to focus on those papers that deal with a single area of activity.

Eighty documents devoted their research to the validation and/or ascertaining of different types of hardware, software or both, in the following areas: prevention, care, diagnosis and treatment.

**Figure 5.**
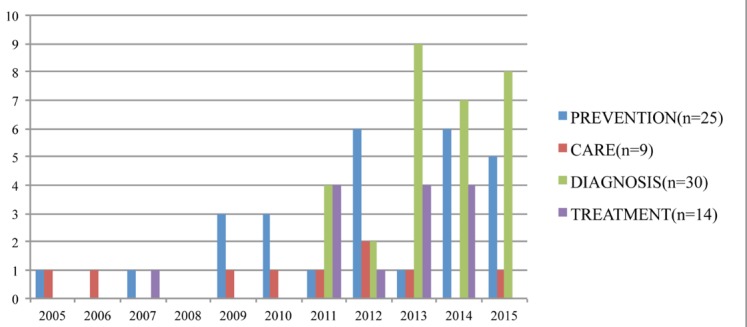
Results found for selected areas when reviewing 2005-2015 and yearly results and selected areas found for the review.

Of the documents reviewed, most were geared to researching and developing hardware and/or software related to diagnosis and prevention of frailty. To a lesser extent, they focused on treatment and care (Fig. 5).

Despite taking into account results that date back to 2005, it was really between 2011 and 2015 during which the greatest number was concentrated (Fig. 5).

### RESULTS

#### 1. Area: Diagnosis

**Figure 6.**
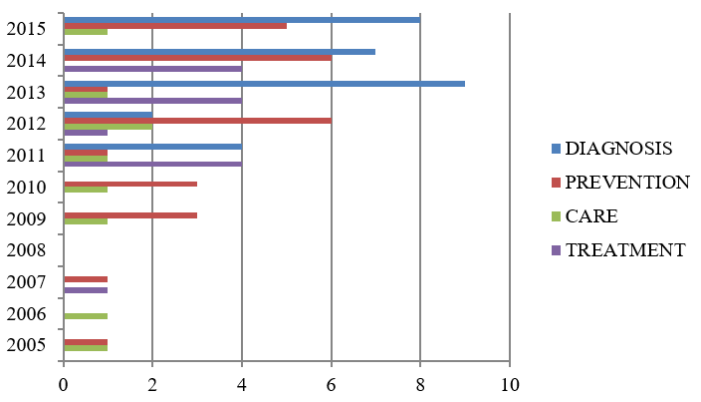
Results found by year and by area: Diagnosis, Prevention, Care, Treatment.

The first studies aimed at researching into some tool or device that might help to diagnose frailty did not appear until 2011 (Fig. 6).

On the one hand, papers were found that focused on an assessment of frailty taking into account the trial-based model.

Ganea et al., 2011[29] conducted a study showing the parameters obtained via a small inertial sensor and portable data recorder (Physilog^®^, BioAGM, CH) fitted to the waist, to distinguish between elderly subjects with differing states of health and functional states.

Martínez-Ramirez et al., 2011[30] carried out a study, the purpose of which was to examine the orientation and acceleration of signals deriving from a triaxial inertial magnetic sensor during balancing trials conducted while the subject was standing up, among a frail, pre-frail and healthy population. The wavelet transform was used in data analysis [31]. The authors concluded by stating that the absolute sum of the wavelet coefficients of the details corresponding to orientation signals and acceleration were associated with the frailty syndrome.

Chang et al., 2013 [32] conducted research, the purpose of which was to integrate wireless sensors and artificial neural networks in order to develop a system capable of gathering data and administering the information required to assess frailty as automatically as possible. To do so, they used a measuring device based on household goods in daily use, with a view to providing home-based means of measurement and thus ensuring that health controls would not be confined to healthcare establishments. The system consisted of five parts: (1) eScale: a scale for measuring the subject’s reaction time; (2) eChair: a chair used to detect slowness of movement, weakness and weight loss; (3) ePad: to measure the subject’s capacity for balance; (4) eReach: to measure functional scope; (5) electronic questionnaire: to measure tiredness when performing an activity; (6) Information base portal: a system based on information obtained from the gateway, so as to gather together all the data and predict the subject’s frailty. The analytical model proposed using an artificial neural network might effectively and easily estimate the level of functional decline. In the long-term, the variation in indicators monitored might permit early detection of frailty and, hence, its early treatment.

Fontecha et al., 2013[33] took advantage of the features and capacities of the mobile phone (accelerometer sensors, capacity for wireless communication and processing capacities, among others) to develop a new method that achieved an objective assessment of frailty in an elderly population - a model with several mobile tools for assessing frailty, that would permit mobility in clinical environments and obtain assessments at any time.

Galan-Mercant et al., 2013; Galan-Mercant et al., 2014 and Galan-Mercant et al., 2015[34-36] managed to identify a series of cinematic variables that demonstrated greater accuracy in discrimination in terms of functional capacity among two groups of elderly persons (frail and non-frail) in phases of the expanded trial involving standing up and leaving (ETUG), using inertial sensors integrated into the iPhone 4^®^. They reached the conclusion that the cinematic parameters obtained from the internal inertial sensors in the iPhone 4^®^ proved promising for the purpose of carrying out the ETUG analysis and that there were encouraging signs insofar as these parameters in separate phases of the ETUG procedure might offer the chance to improve discrimination between frail and non-frail individuals. However, a further in-depth study was still needed to verify the findings.

Zaffarana et al., 2014[37] presented their work at the 10th International Congress of the European Union Geriatric Medicine Society - Geriatric Medicine Crossing Borders, in which they implemented a specific algorithm in the ETUG trial that helped to identify frailty more accurately using inertial sensors integrated into a Samsung Galaxy SII/III.

Castro et al., 2015[38] designed an application known as InCense in which different technologies were found to be involved and integrated into smartphones such as an accelerometer, gyroscope, digital compass, camera, Bluetooth, proximity sensors, GPS, microphones and WIFI. The application gathered together the physical activity carried out by individuals and transferred the resulting information in order to identify frailty.

Papers were also found that focused on the assessment of frailty by taking into account one or more of Fried’s criteria [39].

Gallego et al., 2011[40] presented a study at the 2011 Annual Scientific Meeting of the American Geriatrics Society, the purpose of which was to assess the strength of manual pressure as a predictor of mortality within 6 months in elderly persons following hospitalization as a result of serious illness. A hand-grip electronic dynamometer was used, and the aim was to ascertain whether pressing strength is linked to frailty insofar as it increases mortality. 387 elderly persons over 80 years of age took part. The conclusion was drawn that frailty was linked to a greater vulnerability towards factors involving stress, functional decline and inaccurate health diagnosis. It was shown that a frailty marker such as grip strength (pressing strength) was closely associated with mortality 6 months following hospitalization as a result of serious illness.

Chang et al., 2011[41] designed research using wireless sensors, the purpose of which was to develop a system to detect and assess frailty via interactive multimedia games that would enable users to gather and administer personal information automatically at home. The interactive games incorporated an electronic pressing strength sensor and an electronic pressure sensor that gathered information. They were compared to traditional methods used to measure frailty so as to put their validity and reliability to the test, and they concluded that the device accurately measures grip strength to help detect frailty.

Zavala et al., 2012[42] conducted a study, the purpose of which was to design and assess video games that would enable muscle strength to be measured in order to help detect the first signs of dynapenia (age-related loss of muscle strength). To do so, they created a device that could be incorporated into the remote control of the Wii™ or Xbox 360 console that would measure grip strength. They designed two games with which to measure grip strength and compared this to the traditional measuring method for grip strength using a dynamometer. They concluded that the device accurately measures grip strength and thus helps to detect the first signs of dynapenia - a criterion included in the assessment of frailty.

Chkeir et al., 2013[43] developed a device based on the Grip Ball dynamometer patented in 2008 by Hogrel &Duchêne et al., 2008[44], whereby they more accurately measured grip strength and were able to transmit the data obtained via Bluetooth to a PC or tablet. The results obtained helped to assess the degree of frailty, and they drew the conclusion that the device needed to be improved by carrying out studies on the relevant population in order to obtain a range of strength between sexes.

Hewson et al., 2013[45] proposed the design of an innovative system in order to objectively quantify the level of frailty based on a series of remote tests, each of which use objects similar to those found in homes. A modified ball was used to assess maximum grip strength, while a smartphone equipped with triaxial accelerometer was used to estimate walking speed and the level of physical activity. Lastly, they used a set of bathroom scales to assess involuntary weight loss. All the data generated was then transferred via smartphone to a remote service provider in which the user, their environment and any authorized healthcare professional could access them.

Jaber et al., 2013[46] submitted the ARPEGE at the 2013 IEEE 15th International Conference on e-Health Networking, Applications & Services-Healthcom. This project consisted of a set of technological tools referred to as the ARPEGE package to assess frailty in elderly persons within their habitual environment, i.e. in their home, with reference to Fried’s physical frailty scale [39]. The ARPEGE package comprised different measuring devices connected wirelessly to a tablet-PC that would enable non-professionals to handle them easily. Frailty assessment was of a maximum 8-minute duration, and an initial experiment involving 150 subjects was set in motion at the time the project was submitted. The aim was to demonstrate the acceptability and usefulness of the set of tools.

Dapp et al., 2013[47] presented a study at the 9th International Congress of the European Union Geriatric Medicine Society (EUGMS) that was carried out using the GAITRIte^®^ system for analyzing walking speed that incorporates some wireless sensors attached to the body, whereby objective data about participants’ walking speed was obtained. This objective data was added to other information obtained regarding leisure activities, health-related events, socio-economic, medical and professional aspects, lifestyle habits, limitations in terms of day-to-day activities, mobility problems and risk of falling. The results obtained were then assessed according to Fried’s frailty phenotype [39], and the results showed that the system had sufficient functional competence for the purpose of detecting pre-clinical signs of functional decline.

Drubbel et al., 2013 [48] carried out a study to assess frailty in a cohort of 1580 subjects over 60 years of age in primary care, to compare the results obtained between the Groningen Frailty Indicator (GFI) questionnaire and the FI score calculated previously by researchers, and using software designed by the authors in a prior study [49]. The FI and GI results were moderately superimposed in identifying frailty in elderly community results. Based on the results, the authors suggested estimating an initial FI with recorded routine health data as a means of improving the identification and optimization of resources in primary care. Only patients with a high FI score - i.e. with a high risk of frailty - completed the GFI questionnaire.

Toosizadeh et al., 2014; Toosizadeh et al., 2015 and Toosizadeh et al., 2015 [50-52] implemented a new method to objectively assess frailty using a wireless sensor connection and movement of the upper extremities. Subjects bent their elbow repeatedly for 20 seconds on each side. It was shown that frailty and pre-frailty can be predicted to 94% sensitivity and 98% specificity if compared to Fried’s criteria. The physical assessment is easily made in less than 1 minute.

##### 1.1 Conclusions Area: Diagnosis

A general frailty rate using technology is still to be created. However, we have been able to ascertain that a great deal of research has been carried out since 2011 and is still being carried out to establish the most suitable tool. In the papers reviewed, it has been ascertained that despite isolated developments to measure a variable such as pressing strength or walking speed, the integration of different measuring devices into a single tool is essential for establishing a comprehensive assessment method in which the 5 criteria described by Fried are taken into consideration. From the studies analyzed in this area, the one that gets closest is the ARPEGE project presented by Jaber et al., 2013[46], at the IEEE 15th International Conference on e-Health Networking, Applications & Services-Healthcom. Table 1 contains the studies analyzed.

**Table 1:** Results in area: Diagnosis

Author	Year	Country	Clinic Group	Control Group	Age (Years)	Diagnosis	Area	Method	Classification
Ganea et al., 2011[29]	2011	Switzerland	79	27	≥65	Frailty	Diagnosis	Portable inertial sensor	Hardware
Martínez-Ramirez et al., 2011[30]	2011	Spain	32	24	75-83	Frailty	Diagnosis	Triaxial inertial guidance sensor	Hardware
Chang et al., 2013[32]	2013	Taiwan	160	149	≥65	Frailty	Diagnosis	Wireless sensors and artificial neural networks integrated	Hardware Software
Fontecha et al., 2013[33]	2013	Spain	20	--	78-86	Frailty	Diagnosis	Accelerometer sensor integrated into smartphone	Hardware Software
Galan-Mercant et al.,2013;2014 y 2015[34-36]	2013	Spain	30	--	≥65	Frailty	Diagnosis	Inertial sensor in iPhone4^®^	Hardware
Zaffarana et al., 2014[37]	2014	Italy	94	--	65-90	Frailty	Diagnosis	Inertial sensors in Samsung Galaxy SII/III	Hardware
Castro et al., 2015[38]	2015	Mexico	15	--	73-79	Frailty	Diagnosis	Incense application in smartphone	Hardware Software
Gallego et al., 2011[40]	2011	Spain	387	--	≥80	Frailty	Diagnosis	Hand grip	Hardware Software
Chang et al.,2011[41]	2011	Taiwan	160	149	≥65	Frailty	Diagnosis	Electronic pressure grip force and distance	Hardware Software
Zavala et al., 2012[42]	2012	Mexico	11	--	65-85	Frailty	Diagnosis	Wii™ console using remote sensor	Hardware Software
Chkeir et al., 2013[43]	2013	France	360	--	Senior	Frailty	Diagnosis	Grip-ball Dynamometer	Hardware Software
Hewson et al., 2013[45]	2013	France	--	--	Senior	Frailty	Diagnosis	Grip-ball Dynamometer Digital bathroom scale Acelerometer	Hardware Software
Jaber et al., 2013[46]	2013	France	150	--	≥75	Frailty	Diagnosis	ARPEGE Project	Hardware Software
Dapp et al., 2013[47]	2013	Switzerland	3326	--	≥60	Frailty	Diagnosis	GAITRite^®^-System	Hardware Software
Drubbel et al., 2013 [48,49]	2013	Netherlands	1580	--	≥60	Frailty	Diagnosis	Software GFIdata	Software

#### 2 Area: Prevention

Most results in this area focused on the design of devices and tools aimed at detecting situations of risk and/or falling.

The increase in results was significantly greater after 2012 (Fig. 6).

Lee et al., 2005[53] conducted research that involved placing some cameras on the ceiling of a room fitted out for the study with bedroom furniture such as a bed and chair, among others. The researchers asked the study group to adopt five positions and repeat them three times, and the results of the behavioural pattern were then compared to previous recordings that simulated falls. The system detected 77% of falls and lost 23%. There were only false alarms on 5% of occasions.

Reeves et al., 2007[54] carried out a study in which they used 20 environmental sensors fitted in a home that detected participants’ daily activity with a view to designing algorithms to establish normal behavioural patterns among users - and thus be able to identify any deviations from these normal patterns in real time. When they detected deviations, an alarm went off to alert carers of a possible situation involving risk.

Jun et al., 2009[55], used the 3D VICON movement capturing system and six markers, and to this system they added accelerometers and gyroscopes. The purpose of the study was to obtain detailed parameters in the laboratory that could then be used to detect falls. They studied walking patterns based on silhouettes taken from sequences of images. Three features of the walking patterns were researched from three different image capturing perspectives: shoulder height, spine tilt and centre of the silhouette. By assessing fourteen sequences of images that represented a range of healthy walking styles in frail individuals, features were extracted and compared to the results obtained when capturing movement using the 3D Vicon system.

Zouba et al., 2009[56] used cameras and sensors but incorporated them in a laboratory environment known as GERHOME comprising four rooms: kitchen, living room, bedroom and bathroom. The aim was to conduct a more exhaustive analysis of movement-related patterns in daily life in the search for changes in behaviour that might predict risky situations. The data obtained from 2 volunteers was analyzed (of 64 and 85 years of age), and the accuracy in recognizing postures and events ranged from between 62 and 94%, while sensitivity was within the 62-87% range.

Tolkiehn et al., 2011[57] included an ADXL330 triaxial accelerometer, a barometric pressure sensor (VTI SCP 1000-D01) and a wireless sensor network (BSN) in their research, located in a device attached to the waist. The experimental results showed not only a reliable fall detector but also managed to ascertain the direction of fall, thus being able to predict the location of the affected joint.

Menelas et al., 2012[58] designed a game that would enable users to keep their balance on five different types of flooring (broken stone, stone powder, sand, concrete and wood). The proposed game combined elements from the real world with an interactive virtual one provided by a Kinect™ sensor. When exposing the user to various destabilizing events (disturbances) provided by an interactive shoe, the purpose of the game was to strengthen the lower extremities and prevent falls. On the other hand, as a result of using interactive footwear, it was possible to record users’ dynamics and their capacity to maintain postural stability following a disturbance in real time.

Nakajima et al., 2012[59] carried out a study with the aid of some manipulated inner soles in which the results revealed that capturing walking pattern features may identify elderly persons with a high risk of falling.

Tchalla et al., 2012[60] simply ascertained that a system for switching a light on around the bed significantly reduced falls in the home when the subject placed their foot on the floor when getting in or out of bed, alongside an intercommunication system based on a medal-shaped switch used for assistance in the event of emergency.

Sadasivam et al., 2014[61] proposed the development of a remote-controlled robot (Spykee) equipped with video camera and different sensors that passed over all types of flooring, including carpets. The purpose of the study was to see whether the robot was able to make an assessment of risks in the home by detecting potential hazards and preventing falls.

Ando et al., 2015[62] carried out a laboratory study in which they used all possible capacities of a smartphone to not only detect the risk of falling but also to discriminate between the different types of fall in frail individuals. The fall detection methodology was based on an acceleration and orientation analysis, and the information gathered by the smartphone sensors was processed via threshold algorithms (TAS). The authors showed that the methodology developed was able to detect and classify three types of possible fall (forwards, backwards, seated, on stairs and sideways). This work could be used both for indoors and outdoors in places such as museums, hospitals and public spaces, but also in the home (e.g. for monitoring patients who have recently been discharged from hospital).

Chaccour et al., 2015[63] developed a Zimmer^®^ frame equipped with acoustic signals, infrared sensors, an ultrasound sound, optic sensors and inertial accelerometers. The Zimmer^®^ frame was validated in the laboratory with 5 students, and the results obtained showed the ability of the Zimmer^®^ frame to detect obstacles by giving off an acoustic signal - very useful for preventing knocks and falls.

Dubois et al., 2015[64] proposed a system for preventing falls in the home. This system was based on an in-depth analysis of images provided by the Microsoft Kinect™ sensor, and was designed to detect whether the person being monitored has fallen or is performing a risky activity such as getting on a chair. The results obtained from previous research by Dubois et al., 2013 and Dubois et al., 2014 [65,66] showed that it was possible to identify a person’s activity and to analyze measurements of the walking parameters from in-depth images, although there was a problem with identifying the person being monitored. To recognize the person being monitored, the walking parameters of that person were used rather than facial recognition and algorithms were developed to enable the sensor to detect this, thus managing to personalized prevention of falling in the home without the need for them to carry any other type of device.

##### 2.1 Conclusions Area: Prevention

Preventing risky situations and preventing falls in elderly persons is of the utmost importance so as to thus in turn prevent any increase in their degree of frailty and ensure they remain independent for as long as possible.

With this aim in mind, we found devices in most of the studies reviewed - specifically wireless sensors of different types (motion, optic and pressure, etc.) that determine the risk of falling in elderly persons according to the data recorded and compared to normal behavioural patterns.

In terms of new features, we found on the one hand the inclusion within the research analyzed of something as common in our day-to-day lives as smartphones and, on the other, recognition of the person being monitored from among others who may share the household as obtained using the Microsoft Kinect™ sensor. Lastly, the use of robots constitutes a challenge that is already starting to bear fruit. Table 2 contains the studies analyzed.

**Table 2:** Results in area: Prevention

Author	Year	Country	Clinic Group	Control Group	Age (Years)	Diagnosis	Area	Method	Classification
Lee et al., 2005[53]	2005	Canada	21	--	20-40	Frailty	Prevention	Videocameras	Hardware Software
Reeves et al., 2007[54]	2007	United Kingdom	-- 21 municipios	--	Senior	Frailty	Prevention	20 Wireless environmental sensors	Hardware Software
Jun et al., 2009[55]	2009	USA	--	--	Healthy young adults	Frailty	Prevention	Vicon 3D system 6 markers	Hardware Software
Zouba et al., 2009[56]	2009	France	2	--	64-85	Frailty	Prevention	GERHOME: Cameras and sensors	Hardware Software
Tolkiehn et al., 2011[57]	2011	United Kingdom	12	--	$\bar{x}26$	Frailty	Prevention	Acelerometer Barometric pressure sensor	Hardware Software
Menelas et al., 2012[58]	2012	Canada	--	--	Only laboratory tests	Frailty	Prevention	Kinect™ Interactive shoe	Hadware Software
Nakajima et al., 2012[59]	2012	Japan	498	--	$\bar{x}74$	Frailty	Prevention	Manipulated inner soles	Hadware Software
Tchalla et al., 2012[60]	2012	France	96	98	≥65	Frailty	Prevention	Telecare	Hardware Software
Sadasivam et al., 2014[61]	2014	USA	9	--	71-90	Frailty	Prevention	Robot with videocamera	Hardware Software
Ando et al., 2015[62]	2015	Italy	10	--	25-44	Frailty	Prevention	Smartphone	Hardware Software
Chaccour et al., 2015[63]	2015	France	--	--	Young adults in laboratory	Frailty	Prevention	Optical sensors Inertial acelerometer Audio notification module Location module Infrared sensors Ultrasonic sensor	Hardware Software
Dubois et al., 2015[64]	2015	Switzerland	12	--	21-54	Frailty	Prevention	Sensor Kinect	Hardware Software

#### 3 Area: Care

The number of results in this area has been very stable over most years since 2005, with there being a significant upswing in 2012 (Fig. 6).

Savenstedt et al., 2005[67] established an Internet protocol with broadband together with a conference system, the purpose of which was to set in motion a telecare service. In the results they found technical limitation in transferring communication, and they thought it necessary to carry out further studies in which the technical conditions could improve so as to quantitatively and qualitatively assess the results obtained.

Finkelstein et al., 2006[68] developed the VALUE program in which they introduced the use of an Internet portal that required a PC and video conferencing. The results obtained from this study successfully showed messages being sent and requests for product and care.

Vincent et al., 2006[69] ascertained that the telecare system proved to be far more effective and efficient when the service included healthcare professionals.

Savolainen et al., 2008 and Magnusson et al., 2012 [70,71] presented the ACTION project in which they introduced information and communication technology (ICT) by developing a study with Internet- and video conferencing-based education programs. This program successfully obtained the telecare service and communication among frail elderly persons.

Lin et al., 2008 [72] carried out a study in which they used a physiological wireless sensor system. These biosensors were monitored remotely via WiFi, radio frequency and Universal Plug and Play (UPnP) technology. The results showed early detection of signs of decline, thus improving the quality of care and satisfaction of frail elderly persons.

Mahoney et al., 2009 [73] developed the implementation of wireless sensors based on the ZigBee system in the home via the AT EASE program, to promote care and environmental monitoring and responding to the demand for safety and wellbeing of elderly persons, family members and carers at residential care homes without violating privacy. The results pointed to a perception of the need and usefulness for residents of the system in order to retain their independence and avoid being transferred to a more restrictive environment such as a hospital, this being the key to the program’s success.

Vacher et al., 2011 and Vacher et al., 2013 [74,75] presented the SWEET-HOME audio technology-based smart home project. The results showed the detection of sound, movement and speech in real time - very promising for frail elderly persons with mobility difficulties.

Robben et al., 2012 [76] implemented an innovative portal that encompassed e-health technologies - an information portal about health and wellbeing (ZWIP). The results showed a very important medium for overcoming fragmentation of health care and for facilitating the participation of frail elderly persons. However, it was found to be limited insofar as it has so far been adopted very little in daily practice.

Pigini et al., 2012 [77] implemented an SRS Mobile platform in the SHADOW multi-mission robot, whereby they managed to get the robot to accompany the frail elderly person in performing a range of daily tasks. The results concluded that robot care was positive in the scenarios involving daily life that had been experimented with, except for in the kitchen. Having said this, participants pointed out that interaction with professionals and/or carers offers greater independence.

Clarke et al., 2013 [78] developed an expandable modular platform comprising integrated sensors: four physiological sensors and three environmental sensors. They did not submit any results because the study was still underway. They revealed the fact that initial data was encouraging for the purpose of providing care and detecting possible hazardous events in frail elderly persons.

De Folter et al., 2014 [79] presented the InCasa project that was defined as an integrated network for care/independence of frail elderly persons. The system comprised a sensor for use in bed and two motion sensors, and the results demonstrated the system’s capacity for monitoring daily mobility - very useful for detecting hazardous events and signs of decline.

Man et al., 2015 [80] presented an application platform via interactive software with eleven functions that can be used from any PC, tablet or smartphone. The results showed it to be a very useful and suitable platform for any device, and participants highlighted the fact that it was easy to use as well as being useful as support for their independence and self-sufficiency.

##### 3.1 Conclusions Area: Care

Technology is able to supervise indicators referring to state of health, provide warnings about events such as falls, and give early warning of potential problems and signs of decline in frail elderly persons.

There is also broad recognition of technology’s potential for improving the safety and independence of frail elderly persons. This technology enables quality services to be accessed and the ability for such individuals to remain in their own homes to be extended, thus improving their quality of life by improving their independence.

Technology complements the care work carried out by carers and healthcare professionals but does not yet replace it, because from the previously-described results we can deduce that interaction with professionals and/or carers offers still greater independence. Table 3 contains the studies analyzed.

#### 4 Area: Treatment

This is the area in which the least number of results were found in the course of the present review.

The years in which the greatest number of results were concentrated were as in the case of the Diagnosis area, i.e. between 2011 and 2015 (Fig. 6).

Ganea et al., 2007 [81] carried out a study using a system of inertial sensors attached to a trunk with a data recorder to monitor the activity. The results indicated that the tool was simple yet accurate for the purpose of monitoring frail elderly persons and objectively assessing the effectiveness of a rehabilitation program.

Bondoc et al., 2011 [82] presented the study they had embarked on at the American Congress of Rehabilitation Medicine (ACRM)-American Society of Neurorehabilitation (ASNR) Annual Conference, Progress in Rehabilitation Research. The purpose of this study was to determine the effect of the Nintendo^®^ Wii™ console based on functional interventions regarding the participation and physical condition of frail elderly persons within an institutional environment. No results were obtained because the study was still underway.

**Table 3:** Results in area: Care

Author	Year	Country	Clinic Group	Control Group	Age (Years)	Diagnosis	Area	Method	Classification
Savenstedt et al., 2005[67]	2005	Sweden	18	..	Senior	Frailty	Care	Telecare	Hardware Software
Finkelstein et al., 2006[68]	2006	USA	40	40	≥60	Frailty	Care	Telecare: VALUE program	Hardware Software
Vincent et al., 2006[69]	2006	Canadá	38	--	≥65	Frailty	Care	Telecare	Hardware
Savolainen et al., 2008 y Magnusson et al., 2012[70,71]	2008	Sweden	--	--	Senior	Frailty	Care	Technology of the information and communication. (TICs)	Hardware Software
Lin et al., 2008[72]	2008	Taiwan	--	--	≥60	Frailty	Care	Wireless physiological sensors	Hardware Software
Mahoney et al., 2009[73]	2009	USA	13	--	$\bar{x}79$	Frailty	Care	AT EASE Project	Hardware Software
Vacher et al., 2011 y 2013[74.75]	2011	France	13	--	$\bar{x}35$	Frailty	Care	SWEET-HOME Project	Hardware Software
Robben et al., 2012[76]	2012	Netherlands	290	--	≥70	Frailty	Care	e-salud web site	Software
Pigini et al., 2012[77]	2012	Italy	63	--	75-91	Frailty	Care	Robot Shadow	Hardware Software
Clarke et al., 2013[78]	2013	United Kingdom	11	--	45-82	Frailty	Care	Integrated sensor platform	Hardware Software
De Folter et al., 2014[79]	2014	United Kingdom	--	--	Senior	Frailty	Care	inCASA (integrated network)	Hardware Software
Man et al., 2015[80]	2015	Netherlands	73	--	≥65	Frailty	Care	Interactive Software with 11 functions	Software

Conversely, Kwok et al., 2011[83] did provide results in the study in which they made a comparison between the active Nintendo^®^ Wii™ program and gymnasium-based standard rehabilitation in frail elderly persons so as to ensure a reduction in falls and also fear of falling. The study was the first randomized test conducted using the Nintendo^®^ Wii™ as a tool for reducing falls and the fear of falling in frail elderly persons, and the results showed that use of Nintendo^®^ Wii™ proved to be more effective than the traditional model as it successfully reduced the number of falls. The program is suitably cost-effective.

Szturm et al., 2011[84] conducted a study in which they used pressure and motion sensors together with a biofeedback screen that included three types of video game. The purpose of the study was to ascertain whether balance training via interactive games would result in better control of dynamic balance compared to a standard physical strength and balance program. The results indicated that (1) the biofeedback screen helped to improve balance; (2) improvement in response when tried out on different surfaces; (3) the movements required to complete the experimental tasks were selected randomly whereby new research is required that would take into account a detailed design of the movements to be made; (4) the experimental tasks had levels of difficulty that could be manipulated so as to ascertain and challenge the performance of each individual. The authors plan to carry out future home-based studies.

Daniel et al., 2011[85] presented a study using the Nintendo^®^ Wii™ at the 2011 Annual Scientific Meeting of the American Geriatrics Society, in which they compared the activity carried out by the Geri-Fit^®^ exercise program to that done with the Nintendo^®^ Wii™ and to a control group. The results indicated an improvement in the physical and muscular state of pre-frail participants both in terms of treatment using Geri-Fit^®^ and the Nintendo^®^ Wii™.

Daniel et al., 2012[86] conducted a study in pre-frail elderly persons in which the Nintendo^®^ Wii™ console was combined with exercises performed in the seated position and a control group. The purpose of the study was to ascertain the effectiveness of a new rehabilitation program using some popular games while the participant was wearing a waistcoat laden with weight, thus enabling the degree of frailty of pre-frail participants to be reduced. The results pointed to the fact that there was no difference between intervention groups, and the authors concluded by stating that the exercise program using Nintendo^®^ Wii™ was as effective as the exercise program performed in the seated position. They also stressed that use of the console could prove to be very useful in rehabilitation at home following discharge from hospital and/or to perform the exercise in a group.

Tsai et al., 2013 [87] carried out a study, the purpose of which was to assess the acceptability of an aptitude test application (iFit) in a game environment for installation in an assisted community. The games are based on trials that also serve as the following tests: grip strength; balance and reaction time. It was ascertained that the platform could be used to promote health and prevent the appearance of frailty, and the application could be installed both on PCs and tablets and smartphones.

Jorgensen et al., 2013 [88] conducted a study to examine postural balance and muscle strength in elderly persons from the community. In the course of the study they compared the intervention group to another control group that were wearing ethylene vinyl acetate (EVA) inner soles for daily use. The results indicated significant improvements in maximum muscle strength of the leg and in overall functional performance. Bilateral static postural balance remained unaltered.

Kim et al., 2013 [89] carried out an unsupervised virtual reality study that consisted of a muscle strength exercise program for the hip and balance control. The clinical group evidenced significant improvement in the trials conducted in relation to the control group. The authors insisted that a virtual reality-based exercise program might prove to be a useful tool for improving the reduction in physical function in elderly persons as a home-based exercise, provided that this is supervised.

Lauritzen et al., 2013 [90] took part in the GameUp: Game-Based Mobility Training and Motivation of Senior Citizens project with a work in which they researched into the use of the FitBit Ultra application as opposed to the Samsung Galaxy S3 pedometer smartphone application and some video cameras. The results showed that the FitBit Ultra application was advisable for carrying out physical activity such as walking in young adults and in more elderly persons who need a little technical assistance with walking (stick). However, this application was not considered advisable if a Zimmer^®^ frame is used for walking.

Padala et al., 2014 [91] presented a study at the 2014 Annual Scientific Meeting - American Geriatrics Society in which they conducted a retrospective review of 400 patients ≥ 60 years of age who had undergone rehabilitation in a specialist nursing home. Of these, 63 subjects had the documentation for use of Wii Fit at their disposal during rehabilitation, while a further 63 subjects made up the control group and had no documentation for use of Wii Fit, but only underwent physical therapy. By comparing differences between the groups in terms of the change experienced since the time of admittance in daily activities, balance and the distance covered, the authors concluded that the use of Wii Fit improves these three points, namely balance, the distance covered and daily activities.

Kubicki et al., 2014 [92] carried out a study with a view to researching into the effects of a 2D virtual reality-based program on postural control associated with a swift movement of the arm in frail elderly persons for rehabilitation purposes, by re-learning to use the upper extremities. They used the virtual reality system, Fovea Interactive^®^, and a marker on the arm and forearm. The results suggested that a certain level of motor skill re-learning was retained in frail patients and concluded by saying they thought more training would be necessary to be able to automate the movement.

Geraedts et al., 2014 [93] conducted an assessment study to ascertain compliance with and the effectiveness of an individually-adapted physical activity program undertaken at home for frail elderly persons. This was done using a physical activity sensor in the form of a pendant to ensure it would be portable and would provide remote feedback using a PC tablet on which videos of the exercises were displayed. The results showed that the program constituted an innovative method for stimulating physical activity in frail elderly persons. The authors considered that the insight gained in this study could be used to develop and streamline the application of innovative technology in exercise programs in the home. As a following step, they proposed conducting an effectiveness assessment via a randomized controlled trial.

Geraedts et al., 2015 [94] conducted a randomized controlled trial in which they validated the research carried out in 2014, as described above. In this validation, the authors confirmed that the study group obtained better results in terms of leg mobility than the control group. Therefore, they considered the sensor to be a valuable tool for assessing physical activity in the home based on leg mobility time in frail elderly persons. The authors also thought that further studies would be necessary to specify more specific aspects of walking and postures involved within the daily activity pursued by frail elderly persons.

Fairhall et al., 2015 [95] conducted a randomized controlled trial, the purpose of which was to assess a multi-factor intervention in the development of frailty in pre-frail elderly persons compared to a control group. The results showed mobility improvement, and the multi-factor intervention provided major potential benefits in terms of preventing transition towards frailty.

**Table 4:** Results in area: Treatment

Author	Year	Country	Clinic Group	Control Group	Age (Years)	Diagnosis	Area	Method	Classification
Ganea et al., 2007[81]	2007	Switzerland	30	--	74-86	Frailty	Treatment	Inertial sensor system	Hardware Software
Bondoc et al., 2011 [82]	2011	USA	20	20	Senior	Frailty	Treatment	Wii™ sports and Wii Fit program	Hardware
Kwok et al., 2011[83]	2011	Singapore	40	40	≥60	Frailty	Treatment	Nintendo^®^ Wii™ console	Hardware
Szturm et al., 2011[84]	2011	Canada	14	16	65-85	Frailty	Treatment	Pressure and motion sensors	Hardware Software
Daniel et al., 2011[85]	2011	USA	12	11	≥65	Frailty	Treatment	Geri-Fit^®^ program Wii™ console	Hardware Software
Daniel et al., 2012[86]	2012	USA	16	7	≥70	Frailty	Treatment	Wii Fit Nintendo^®^Wii™ console	Hardware
Tsai et al., 2013[87]	2013	Taiwan	101	--	≥60	Frailty	Treatment	iFit fitness testing platform	Hardware Software
Jorgensen et al., 2013[88]	2013	Denmark	28	30	≥65	Frailty	Treatment	Nintendo^®^Wii™ console	Hardware
Kim et al., 2013[89]	2013	South Korea	18	14	65-72	Frailty	Treatment	Virtual reality	Hardware Software
Lauritzen et al., 2013[90]	2013	Sapin	18	--	81-90 25-45	Frailty	Treatment	Fitbit Ultra Samsung Galaxy S3	Hardware
Padala et al., 2014[91]	2014	USA	63	63	≥70	Frailty	Treatment	Wii Fit	Hardware
Kubicki et al., 2014[92]	2014	France	23	23	≥70	Frailty	Treatment	Based on 2D virtual reality Fovea Interactive^®^ and marker	Hardware
Geraedts et al., 2014[93]	2014	Netherlands	50	--	70-85	Frailty	Treatment	Wireless motion sensor	Hardware Software
Geraedts et al., 2015[94]	2015	USA	20	--	≥70	Frailty	Treatment	Hybrid sensor: acelerometer and barometric pressure sensor	Hardware
Fairhall et al., 2015[95]	2015	Australia	115	115	≥70	Frailty	Treatment	Multifactorial intervention program with online exercises	Interactive computer software

##### 4.1 Conclusions Area: Treatment

Most research activity was concentrated between 2011 and 2015 in the case of this area. Regular physical activity is essential for elderly adults in general, as this is considered to be the way to remain healthy and independent.

The studies mainly used the Nintendo^®^ Wii™ console to promote physical activity. The putting into practice of this type of console has thus far been tried out in the area of rehabilitation, although its use is confined to games designed for the console and these games do not always meet the full requirements of certain types of treatment.

A further step forward is offered by the introduction of virtual reality using the Fovea Interactive^®^ system as a re-learning method.

Different mobile applications such as FitBit, iFit or some other type of motion sensor or activity gauge both promote and improve physical activity such as walking. This might be used to assist other technologies so as to form part of a more integral method.

The multi-factor intervention program is very complete, but requires the intervention of many healthcare professionals that means its accessibility is limited. Table 4 contains the studies analyzed.

## DISCUSSION

This paper provides a review of the most relevant devices and technologies that were developed between 2005 and 2015 in the different areas covered by frailty, namely prevention, care, diagnosis and treatment. In most of the results, classified according to areas, the objective that had been set out for each study was met, and all studies show that technologies make it possible to work in different areas linked to frailty.

However, what is the most suitable technology for each area? May we conclude by stating that these technologies are genuinely useful in each area?

After having analyzed each study one by one, it is important to focus attention on the set of these technologies classified according to areas and to compare them, so as to find a satisfactory response to these questions.

In the area of diagnosis, the scales on which the authors based the design of the devices are: Fried’s phenotype model of frailty and the trial-based model. 15 studies stand out among those that assess frailty, taking into account the different models described.

**Figure 7.**
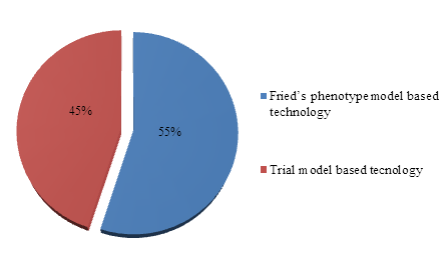
Percentage of results according to the scales on which the authors base for the design of technology in the area: Diagnosis.

This review shows how research provides very similar results in terms of the development of technologies based on one scale or another (Fig. 7).

Irrespective of the scale used for the design, advances made in wireless technology can be noted, as can the integration of smartphones, within the interactive multimedia game world - and even in smart clothes that may facilitate more standardized assessment in daily physical activity [96].

Thus, when assessing the functional capacity of elderly persons, healthcare professionals may have objective information at their disposal for assessment purposes, as the information provided is often based on self-reports by the interested parties themselves, and this may vary a great deal from one individual to the next.

**Figure 8.**
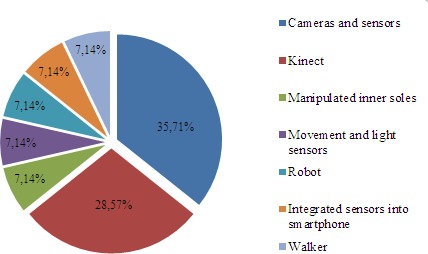
Percentage of devices used in studies for fall prevention.

The results show that there is a correlation between the frailty results obtained via the interactive game system and the results obtained from traditional measuring methods. They also show that the interactive game-based system is a predictive tool of great specificity that can be used to assess frailty.

The most recent finding related to sensors attached to the arm and forearm features, in addition to its simplicity, a unique characteristic that is its applicability in individual health clinics. To ascertain test-retest reliability and the viability of a new method, the authors envisage assessing the tool in a larger sample, in different groups of individuals and in different areas of health care.

These parameters might in the future prove to be of great interest in the clinical sphere of activity to help with methods used to identify the elderly population with frailty syndrome.

In the area of prevention, frailty is also recognized as a factor involving risk of falling. The negative consequences related to falls include fear of falling, loss of confidence, anxiety, depressive symptoms and reduction in self-sufficiency, which may lead to social isolation and/or avoidance of physical activity. However, it is also known that up to 40% of falls can potentially be prevented, according to Kojima et al., 2015[97].

This review shows how research provides very similar results in terms of the development of devices that use both wireless sensors with cameras and Kinect™ sensors to analyze movements and postures that may indicate a risk of falling (Fig. 8)

Promising results have been obtained for future studies that may increase the accuracy both of extracting features of risky patterns and postures such as the monitoring of elderly persons thought to be at risk.

Using a robot to identify possible risks in the home is a new feature, although the study itself pinpoints to several research questions about how best to use remote-controlled robots in another sense that may for instance help to reduce the number of home visits by staff and therefore improve efficiency owing to cost reduction.

**Figure 9.**
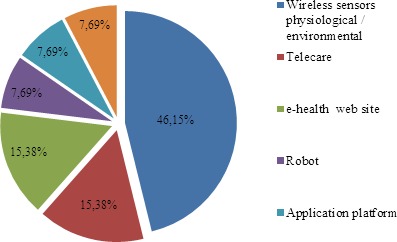
Percentage of tools used in the study for care.

In the area of care, the highest percentage was found in the use of different wireless sensors - motion, physiological and environmental in particular - that constitute what are known as smart homes based on different back-up technologies in terms of software needed to develop them. They can prove very interesting in the area of care for frail elderly persons in order to encourage their independence and also improve their quality of life. According to what the authors have been able to ascertain, telecare would seem to be very effective and efficient when the service involves professionals. The application platform is a new system that uses an interactive software that can be operated via any device such as a tablet or smartphone - a feature that encourages independence of frail elderly persons, thus increasing their quality of life. Conversely, the use of robots in care has not yet gained popularity, with contact with other individuals such as carers still being preferred (Fig. 9).

In the area of treatment, the Nintendo^®^ Wii™ console is the most-used tool in the studies analyzed for treatment of frailty in elderly persons. The results show better effectiveness over the traditional method or other tools used in each case, but the disadvantage is that when performing the games that are habitually used in studies, only one or two extremities are involved. These are not activities that envisage more complete ones in which both the trunk and the extremities take part in the game.

Although the physical activity program that is adapted individually via remote feedback using a tablet-PC that shows exercise videos proved to be very effective, it is limited by the motion sensor used to record only activity involving the legs. The authors consider that future research could include extending the activity programs at home.

In using pressure and motion sensors with interactive games, the introduction of automatic adaptation of the game being played based on the signals received from the sensors in order to balance the difficulty faced with perceived skills or the physical condition in order to offer better incentives in terms of the player’s participation, is considered to be an interesting improvement that could be taken into account when using systems with such interactive games.

**Figure 10.**
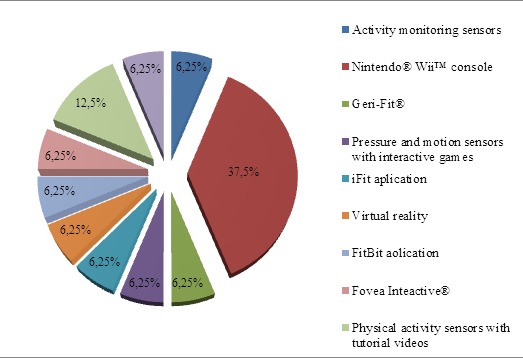
Percentage of devices used in the studies for treatment.

Virtual reality is currently in the developmental phase in terms of treatment of frailty. Only one study provides us with insight about this complex system, albeit one replete with possibilities (Fig. 10).

The studies show that all technologies are suitable for use as tools for treating frail elderly persons.

The serious games appear in 3 of the 4 revised areas. According to the following relation regarding the total: number of articles with use serious games/number of total articles. The relation is: Diagnosis (1/16), Prevention (2/12), Care (0/12) and Treatment (7/15).

At present, the serious games are a tool with many possibilities that are being slowly implemented in frailty researches and from which good results are expected in a near future.

### General conclusions drawn from the review conducted

The application of technological solutions in health care is a field that is constantly expanding and about which there are great expectations both on the part of users and healthcare professionals and carers, despite the existing reticence in this area known as a technology gap.

Worldwide, the number of elderly members of the population and the need to be able to assist them properly is on the increase. This need requires major material and human resources that in turn increase costs.

It is of the utmost importance to continue working to reduce the gap existing between technology, frail elderly persons, healthcare professionals and carers by bringing together the different views about technology and thus stimulate dialogue, an increased awareness and knowledge about the respective fields in order to engage in collaboration on projects that may reduce costs and improve health and quality of life.

The researchers should think not only in searching new tools that include active participation of the subjects, but also that involve amusement in order to obtain considerable implication so that success in participation, adherence and activity compliance is achieved regardless the frailty area in which it is developed. In this way, serious games are very interesting.
